# Exploring the Role of the Gut and Intratumoral Microbiomes in Tumor Progression and Metastasis

**DOI:** 10.3390/ijms242417199

**Published:** 2023-12-06

**Authors:** Aneta Sevcikova, Beata Mladosievicova, Michal Mego, Sona Ciernikova

**Affiliations:** 1 Department of Genetics, Cancer Research Institute, Biomedical Research Center of the Slovak Academy of Sciences, Dubravska cesta 9, 845 05 Bratislava, Slovakia; aneta.sevcikova@savba.sk; 2 Institute of Pathological Physiology, Faculty of Medicine, Comenius University, Sasinkova 4, 811 08 Bratislava, Slovakia; beata.mladosievicova@fmed.uniba.sk; 3 2nd Department of Oncology, Faculty of Medicine, Comenius University and National Cancer Institute, Klenova 1, 833 10 Bratislava, Slovakia; misomego@gmail.com

**Keywords:** gut microbiome, intratumoral microbiota, cancer progression, metastasis, epithelial-to-mesenchymal transition, angiogenesis, microbiota modulation

## Abstract

Cancer cell dissemination involves invasion, migration, resistance to stressors in the circulation, extravasation, colonization, and other functions responsible for macroscopic metastases. By enhancing invasiveness, motility, and intravasation, the epithelial-to-mesenchymal transition (EMT) process promotes the generation of circulating tumor cells and their collective migration. Preclinical and clinical studies have documented intensive crosstalk between the gut microbiome, host organism, and immune system. According to the findings, polymorphic microbes might play diverse roles in tumorigenesis, cancer progression, and therapy response. Microbial imbalances and changes in the levels of bacterial metabolites and toxins promote cancer progression via EMT and angiogenesis. In contrast, a favorable microbial composition, together with microbiota-derived metabolites, such as short-chain fatty acids (SCFAs), can attenuate the processes of tumor initiation, disease progression, and the formation of distant metastases. In this review, we highlight the role of the intratumoral and gut microbiomes in cancer cell invasion, migration, and metastatic ability and outline the potential options for microbiota modulation. As shown in murine models, probiotics inhibited tumor development, reduced tumor volume, and suppressed angiogenesis and metastasis. Moreover, modulation of an unfavorable microbiome might improve efficacy and reduce treatment-related toxicities, bringing clinical benefit to patients with metastatic cancer.

## 1. Introduction

The emerging trend of microbiome research in oncology results from studies uncovering the role of microorganisms in the etiology of several malignancies. Preclinical and clinical studies have also revealed a significant impact of the gut and tumor microbiomes on the efficacy of antitumor therapy and treatment-induced toxicity [1]. Moreover, mounting research focuses on the analysis of the microbiome composition in metastatic disease [2]. The significant role of the microbiome in oncogenesis and treatment underlines the fact that polymorphic microbiomes, including intestinal, oral, skin, tumor, lung, and vaginal microbiomes, were added to the extended comprehensive concept termed “The Hallmarks of Cancer”, which summarizes the key characteristics of tumors. The microbiome directly interacts positively or negatively with other hallmarks of malignancies, such as inflammation, immune impairment, genomic instability, and resistance to antitumor therapy [3].

The study of metastasis biology at the cellular, molecular, biochemical, and physical levels has undergone dramatic growth over the last 20 years. While the precise pathways are still under investigation, recent research has indicated new roles of cancer cells, which involve promoting genes with metastasis-driving mutations, cancer stem cells, circulating tumor cells (CTCs), epithelial-to-mesenchymal transition (EMT), and the metastatic dormancy and dynamic plasticity of cancer cells [4,5]. Various studies also demonstrated that the following drive metastatic spread: systemic inflammation; immune system modulation; specific interactions between cancer cells, immune cells, and cells in the tumor microenvironment; the avoidance of anoikis; immune checkpoint regulation; self-seeding, and other mechanisms. Mounting research highlights the role of the intratumoral and mucosal microbiomes in the progression of metastatic processes.

The mechanisms by which the intratumoral microbiota is implicated in metastatic spread are still unclear [6]. Recently, a newly developed single-cell RNA-sequencing method, known as INVADEseq, aimed to identify cell-associated bacteria in patient tumor samples and describe the pathways involved in metastatic processes [7]. According to the findings, the highly organized distribution of bacteria within tumors affects immune and epithelial cell functions, leading to disease progression [7]. Studies reported that specific microorganisms might affect metastatic processes, including EMT, resistance to fluid shear stress, immune system modulation, and matrix metalloproteinase (MMP) regulation [8]. EMT-related pathways might be regulated by specific microbial pathogens [9,10]. Moreover, the microbiome can change the actin cytoskeleton, contributing to tumor cells’ resistance to fluid shear stress [11]. As previously shown, *Helicobacter pylori* supports the remodulation of actin filaments, which results in EMT onset [12,13]. In addition, specific microbes within the microbiome contribute to tumor cell adaptation to specific biochemical factors in the tumor microenvironment during metastatic processes [8].

In this review, we aim to summarize the current knowledge about the emerging role of the gut and intratumoral microbiomes in metastasis and cancer progression. We analyze studies focusing on the association between the composition of microbial communities and metastatic disease in patients with different types of malignancies. Moreover, we outline the mechanisms of microbiome involvement in cancer progression and metastasis-related processes, including EMT and angiogenesis. Microbiota modulation represents an evolving phenomenon in cancer patient care, and mounting evidence supports the clinical utility of a microbiome-based approach in cancer initiation, progression, and treatment.

## 2. The Mechanisms of Tumor Progression and Metastasis

**Figure 1:**
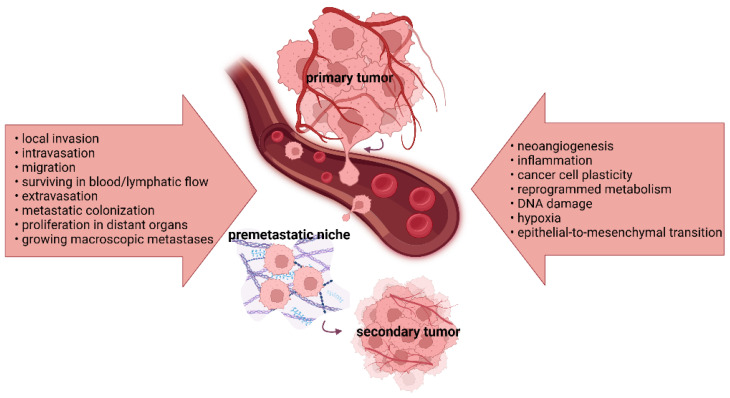
The key processes involved in tumor progression and metastasis. A deep understanding of the crucial events and corresponding mechanisms leading to the formation of distant metastases is essential for developing treatment modalities to target different stages of cancer development and improve patient outcomes.

Tumor progression and metastasis represent multi-step processes, resulting in cancer cell changes that enable them to grow, spread, and establish secondary tumors at distant body sites (Figure 1).

The activation of invasion and metastasis is initiated by epigenetic changes, cell–cell interactions, growth factors, cytokines, signals from extracellular matrix components, extracellular matrix mechanical pressures, and the intratumoral microbiota [14].

The metastatic cascade includes the detachment of cancer cells from the primary tumor and the gaining of an invasive phenotype, local invasion into surrounding tissue, intravasation into the circulation, systemic transportation, extravasation, and the formation of colonies at distant sites, with adaptation and proliferation in secondary organs.

CTCs typically arise from epithelial tumor cells that undergo EMT, resulting in the loss of cell–cell adhesion and apical–basal polarity, the reorganization of the cytoskeleton, acquiring properties of tumor stem cells, and resistance to therapy. This process is regulated by transcription factors in tumor cells (Snail 1, Slug, ZEB1, Twist, FOXC2, etc.) and signaling pathways from the tumor microenvironment (WNT, Notch, Hedgehog, TGFβ, FGF, EGF, HGF signaling, etc.). Additionally, the hypoxia and activation of specific signaling pathways, including PI3K, WNT/β-catenin, and MAPK, affect EMT regulation [15,16]. Many studies focus not only on CTC detection and enumeration but also on CTC biomarkers, among which EMT markers are of great interest [17,18,19]. The most aggressive CTCs are related to the infiltration of the primary tumor or established metastasis in a process of “self-seeding”. Self-seeding in metastasis is the recruitment of cancer cells and the re-seeding of primary tumors and existing metastases by aggressive cancer cell clones [20,21].

Cancer cells can induce neutrophils to release neutrophil extracellular traps (NETs), which sequester CTCs and promote the metastatic process [22,23,24,25,26]. A certain number of CTCs can be eliminated by anoikis, the programmed apoptosis of cells [27]. However, cells can develop an anoikis-resistant state via oncogene activation (e.g., *ERBB2* and *RAS*), an integrin switch (e.g., the downregulation of αvβ3 integrin expression), the constitutive activation of antiapoptotic pathways (e.g., the PI3K/Akt signaling pathway), the triggering of EMT, microRNAs (e.g., the downregulation of the miR200 family), high oxidative stress (e.g., activated growth factor receptors increase intracellular reactive oxygen species production by activating enzymes such as NADPH oxidase and lipoxygenase), hypoxia, the modulation of extracellular matrix stiffness, and the metabolic reprogramming of cancer cells [28]. Tumor cells can attach to specific distant organs/tissues and form colonies through distinct adhesion molecules, including proteoglycans (e.g., CD44), mucins (e.g., MUC16), integrins (e.g., α2β1), and the members of the immunoglobulin superfamily (e.g., ICAM1, VCAM1, and L1CAM) [29].

Before the arrival of tumor cells from primary tumors to the premetastatic niche [30], hematopoietic progenitor cells (VEGFR1-positive) travel from the bone marrow into the circulation and establish themselves in secondary organs, where they adhere to fibronectin, produced by fibroblasts and fibroblast-like cells [31]. The adherence is mediated by the integrin VLA-4, expressed by hematopoietic progenitor cells [32]. The nidation of tumor cells is primarily influenced by stromal-derived factor 1 (SDF-1), binding to the chemokine receptor CXCR4 [33]. CXCR4 receptor expression on breast cancer tumor cells is a typical determinant of bone metastasis [34,35]. Its activation results in pseudopodia formation and integrin modulation, followed by the recruitment of endothelial cells (VEGFR2-positive) to the distant site [36].

Cancer cells and the tumor microenvironment produce factors that influence angiogenic processes, with the key drivers being VEGF-A [37,38] binding to VEGFR2 receptor [39]. Alterations of protooncogenes (*RAS* and *SRC*) and tumor suppressor genes (*TP53* and *VHL*) correlate with VEGF overproduction by tumor cells. Hypoxia is the principal stimulator of VEGF production, and hypoxia-inducible transcription factors (HIF-1α and HIF-2α) play a central role in VEGF regulation. Other angiogenesis inductors, such as FGF1, EGF2, PDGF-B, PDGF-C, and EGF, bind to their respective receptors on blood vessel endothelial cells and induce proliferation and migration [40]. Besides the conventional angiogenic mediators, BMP9 signaling and Shh signaling also participate in the process [41]. In addition, exosomes released by cancer and immune cells may transport various proangiogenic molecules like VEGF, MMPs, and microRNAs [42].

## 3. The Relationship between Microbiome and Cancer Progression-Related Processes

**Figure 2:**
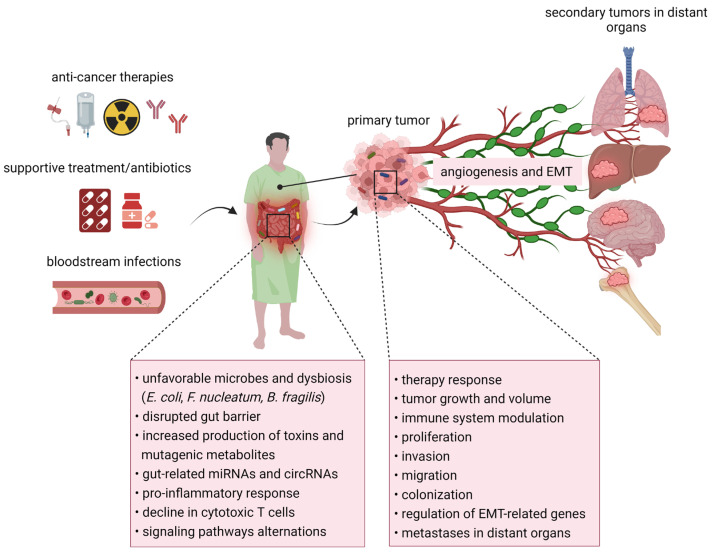
The involvement of the gut and intratumoral microbiome in metastatic processes. Not only cancer development but also the type of anti-cancer therapy affects the diversity of microbial composition in the gastrointestinal tract and alters microbial-associated metabolites. The deregulated thickness of the gut mucosal layer might be responsible for bacterial translocation and development of bloodstream infections. Gastrointestinal dysbiosis results in the inflammation that promotes cancer cell spread due to changed immune responses. Microbes within the tumor microenvironment affect the progression of cancer via modulated immunity and changed inflammatory signaling pathways. Moreover, studies observed the relationship between intratumoral bacteria and metastasis via increased resistance to mechanical stress. Abbreviations: EMT, epithelial-to-mesenchymal transition.

In recent years, the correlation between the microbiome, cancer, and metastatic disease has gained more attention (Figure 2). Many studies confirmed that certain microbes and their metabolites are associated with a better/worse therapy response and patient outcomes.

Understanding the mechanisms by which unfavorable microbes have an impact on tumor progression is an active area of recent research. Therefore, intensive research in numerous ongoing clinical trials might shed more light on prognostic microbial markers for treatment outcomes in metastatic disease (Table 1). The identification of microbial biomarkers will help to understand how the microbiome is implicated in cancer progression.

### 3.1. Microbiome in Epithelial-to-Mesenchymal Transition

The association between specific microbiome compositions, the inflammatory response, and therapy resistance related to EMT is still under debate [43]. Alterations in the gut microbiome might support EMT via the TGFβ, WNT, and Notch signaling pathways, and Slug, SNAIL, Twist, ZEB1, and ZEB2 transcription factors resulted in invasive and metastatic cancer processes [44,45,46]. A pathological microbiome in pancreatic ductal adenocarcinoma (PDAC) patients promoted EMT via the activation of transcription factors, including TGFβ and TNFα [47]. Antibiotic-induced gut dysbiosis supported macrophage activation and the production of inflammatory cytokines, subsequently promoting EMT in colorectal cancer (CRC) [48]. *Fusobacterium nucleatum*, Enterococcus faecalis, Bacteroides fragilis, Escherichia coli, and Salmonella enterica are known microbes involved in CRC progression, producing virulence factors that contribute to EMT and cancer progression [49]. *F. nucleatum*-infected CRC cells supported the EMT cell phenotype and elevated cancer cell migration, as well as tumorsphere formation [50]. Similarly, EMT-related morphological changes and the upregulation of mesenchymal marker ZEB1 were documented in cag pathogenicity island+ *H. pylori*-infected gastric epithelial cells [51]. Marques et al. noted that the *H. pylori* infection of gastric cells reduced Afadin and increased ZEB1, vimentin, Slug, Snail, and N-cadherin. Since Afadin regulates adherens and tight junctions, its loss due to bacterial infection might play a role in aggressive gastric cancer phenotypes [52].

The tumor-associated butyrate-producing bacterium SM4/1, *E. coli str. K-12 substrate MG1655*, *Saccharomonospora viridis DSM 43,017*, and *E. coli O157:H7 str. EC4115* correlated with the expression of EMT-related genes (*TGFB*, *RhoA*, vimentin-associated genes, *SNAI2*, *SNAI3*, and *TWIST1*) in muscle-invasive bladder cancer. In addition, the level of both *E. coli O157:H7 str. EC4115* and *Oscillatoria* sp. *CCAP 1459/13* was negatively associated with E-cadherin expression [53]. In the T24 cell line, infection with *E. coli* significantly upregulated vimentin, reactive oxygen species levels, and stemness markers (CD44, NANOG, SOX2, and OCT4), while it downregulated CK19. The authors observed that *E. coli*-infected bladder cancer cells had a specific elongated morphology with a shortage of contact between cells, while non-infected cells had an adhesive ability with epithelial morphology [54]. Gingival squamous cell carcinoma tissue samples showed the presence of *Porphyromonas gingivalis* [55]. *Porphyromonas* supports the invasion and metastasis of oral cancer cells via EMT processes. The exposure of cancer cells to *P. gingivalis* for a long period promoted migration, invasiveness, and resistance to chemotherapeutics [56,57]. The infection of gingival epithelial cells with *P. gingivalis* increased ZEB1 expression, leading to EMT regulation. Conversely, bacterial strains lacking FimA did not promote the expression of ZEB1 [58].

Serum levels of bacterial lipopolysaccharide (LPS) were found to be elevated in individuals diagnosed with esophageal cancer. An in vitro study by Peng et al. revealed an increase in N-cadherin and vimentin in EC109 cells after treatment with LPS, accompanied by reduced E-cadherin levels. The achieved results suggest the potential role of LPS in promoting migration, invasion, and EMT initiation [59]. Microbiota might control cancer stem cells via miRNA and circRNA regulation [60]. Broad-spectrum antibiotic treatment upregulated the level of mmu_circ_0000730, showing its association with cancer cell stemness and EMT via an upregulated SOX9 expression level. As reported, siRNA targeted mmu_circ_0000730-inhibited invasive/migrative processes and reduced metastasis development [61]. Yan et al. confirmed the involvement of *F. nucleatum* in cancer stem cells and EMT crosstalk in cancer progression. The expression levels of EMT markers (E-cadherin and N-cadherin) and a cancer stem cell marker (Nanog) correlated with the abundance of *F. nucleatum* in CRC tissue samples [62]. Previous data observed that infection with *Fusobacterium* upregulated miR-21 levels, and the upregulation of miR-21 correlated with distant metastases and cancer stage [63]. In breast cancer, bacterial metabolites, such as short-chain fatty acids (SCFAs), lithocholic acid, cadaverine, and indole derivatives, exert an influence on the EMT process [64,65]. Ujlaki et al. screened the antineoplastic properties of bacterial-associated metabolites. The application of 3-hydroxyphenyl acetic acid, 4-hydroxybenzoic acid, and vanillic acid led to a hyperproliferative impact on breast cancer cells, while the application of butyric acid, glycolic acid, d-mannitol, 2,3-butanediol, and trans-ferulic acid had cytostatic effects. Anti-EMT properties were documented in the case of applied 3-hydroxyphenyl acetic acid, 4-hydroxybenzoic acid, 2,3-butanediol, and hydrocinnamic acid [65]. The treatment of breast cancer cells with indolepropionic acid, a microbiota-derived tryptophan metabolite, reverted EMT [66].

### 3.2. Microbiome and Angiogenesis

Tumor angiogenesis is involved not only in tumor growth but also in tumor progression and metastasis. According to findings, microbiota plays a role in angiogenesis via VEGF and inflammatory cells [67]. Studies showed that microbiota-derived SCFAs may have an impact on angiogenesis. Butyrate, a major member of SCFAs, plays a role in many cellular processes. Decreased levels of sodium butyrate supported angiogenesis [68]. Lithocholic acid is a bacterial metabolite, and it plays a role as a tumor promoter in human CRC cells [69]. Enterotoxin produced by enterotoxigenic *E. coli* might activate a cGMP-dependent signaling pathway that decreases VEGF and VCAM-1, both associated with angiogenesis and tumor metastasis [70]. Trimethylamine N-oxide (TMAO), as a microbiome-derived metabolite, increased CRC cell proliferation in vitro. Moreover, in vivo experiments confirmed higher levels of TMAO in the circulation, an increased tumor volume, and promoted angiogenesis in choline-fed CRC mice [71]. Changes in the gut microbiome participate in the transformation of adenomas into carcinomas. These alterations resulted from reduced butyrate levels and the secretion of mutagenic metabolites, including spermidine and trimethylamine. Therefore, the CRC microbiome might promote inflammation, angiogenesis, and apoptosis via increased/decreased histamine, glutamine, and pyruvate levels [72]. The crosstalk between the microbiome, macrophages, and cancer cells is still unrevealed. CRC-derived exosomes can interplay with macrophages within the tumor microenvironment and participate in angiogenesis. Moreover, macrophages activated by microbiome-derived exosomes might induce inflammation [73]. Feces transferred from CRC patients into a model of germ-free and azoxymethane-treated mice upregulated genes associated with angiogenesis, invasion, metastasis, and proliferation [74]. Four quorum-sensing peptides from *Bacillus subtilis* (PhrG), *Streptococcus mitis* (CSP), and *E. coli* (EDF) with its tripeptide analog NWN promoted angiogenesis and breast cancer cell invasion [75]. Similarly, the bacteria-produced quorum-sensing peptides such as EntF-metabolite from *Enterococcus faecium*, Phr0662 from *Bacillus* spp., and EDF derived from *E. coli* stimulated colon cancer cell invasion and angiogenesis [76].

## 4. The Studies of Microbiome Composition in Metastatic Disease

Recently, Hilmi et al. studied samples obtained from the lymph nodes, lungs, and livers of patients suffering from different cancer types, such as breast, lung, and colorectal malignancies. A higher presence of *F. nucleatum* was specific to lung metastases. The microbial load in lymph node metastases was lower than in liver and lung metastases. However, the authors did not observe a relationship between the type of primary tumor and the microbial composition in metastases [77]. The level of *Eubacterium halli* in stool samples is negatively associated with fatigue in patients with advanced, metastatic, unresectable colon, ovarian, cervical, and non-small-cell lung cancers [78]. In vivo experiments confirmed that gut microbial depletion via a broad-spectrum antibiotic cocktail reduced the incidence of metastases in melanoma, pancreatic, or colon cancer murine models [79]. Spakowicz et al. performed a retrospective analysis of 690 patients treated with immunotherapy for metastatic melanoma or non-small-cell lung cancer. The results showed that antibiotics and corticosteroids reduced overall survival (OS), but no direct microbiome measurements were performed [80].

### 4.1. Gastrointestinal Malignancies

Gut dysbiosis and intestinal inflammation represent critical factors in CRC progression [81]. Many studies documented differences in the gut microbiome composition between early and definitive CRC stages [82,83]. Higher levels of *F. nucleatum* in fecal and tumor samples correlated with advanced-stage CRC in patients. This microorganism activated tumor-derived CCL20 expression, increased macrophage infiltration, induced M2 polarization, and enhanced metastasis [84]. Similarly, *F. nucleatum* was more abundant in feces from patients with positive lymph node metastases. In vitro experiments showed that bacterial infection increased the migration of HCT-116 and LoVo cells. However, the heat-killed bacterium could not support cell migration. Accordingly, an in vivo analysis showed that an injection of *Fusobacterium*-treated cells induced lung metastatic nodules in nude mice [85]. *Fusobacterium* might contribute to CRC metastases via declined m^6^A modifications in CRC cells and patient-derived xenografts, which resulted in more aggressive cancer cell phenotypes [86]. The presence of *Fusobacterium* in primary colon adenocarcinoma correlated with *Bacteroides*, *Selenomonas*, and *Prevotella* in distant metastases. Viable *Fusobacterium* was detected in murine xenografts, and metronidazole administration significantly reduced intratumoral *Fusobacterium* load and tumor growth [87].

Wang et al. assessed the effect of combined regorafenib plus toripalimab in patients with refractory metastatic CRC. Patients not responding to combined treatment were reported to have an increased amount of *F. nucleatum*. A lower response rate was documented in patients with liver metastases [88]. However, supplementation with *Faecalibacterium prausnitznii* could reverse CRC progression via several mechanisms and reduce inflammation [89]. In mice, a colonic bacterial biofilm composed of *E. coli* and *B. fragilis* was responsible for faster CRC onset, disease progression, and mortality than colonization only with one bacterial strain alone [90]. The *B. fragilis* toxin is responsible for lost cell adhesion resulting in EMT [91]. Changes in the EMT-associated genes *SNAI1* and *VIM* were documented after the co-cultivation of Caco-2 cells with bacteria from CRC biopsies. The downregulation of *SNAI1* was observed in all affected cells. However, *VIM* expression changes depended on co-cultivation with specific bacteria. *Proteus vulgaris* increased *VIM* expression, but *Proteus mirabilis* and *Pseudomonas aeruginosa* were associated with reduced expression [92]. Sun et al. assessed the effect of the Quxie capsule (QX) as herbal medicine on the gut microbiome in patients with unresectable metastatic CRC. A decreased amount of Actinobacteria became elevated after QX treatment. An LEfSe analysis showed that the microbiome before QX treatment was composed of c_*Gammaproteobacteria* and g_*Lachnospiraceae UCG 008*, while f_*Ruminococcaceae* and g_*Peptostreptococcus* dominated after QX treatment. Moreover, high levels of CD4 and CD8 cells were observed in the QX-treated group [93].

The poor prognosis of pancreatic cancer reflects the necessity of novel non-invasive markers in the management of patients suffering from metastatic or locally advanced pancreatic cancer. Therefore, a fecal microbiome analysis might help to determine potential microbiome-related biomarkers in the early diagnosis of patients [94]. Jeong et al. investigated the microbiome in tumor and normal adjacent tissue samples from patients with PDAC. A higher number of metastases in lymph nodes correlated with a higher abundance of *Comamonas* and *Turicibacter* in tumor tissues, while there was a lower amount of *Tepidimonas*, *Enhydrobacter*, *Turicibacter*, and *Wautersiella* in normal tissues. Alpha diversity was higher in patients with cancer recurrence with low levels of *Streptococcus* and *Akkermansia* in tumor samples [95].

The administration of a nanoparticle conjugation of ginsenoside Rg3 (NpRg3) reduced Firmicutes and increased Bacteroidetes and Verrucomicrobia in the dimethylnitrosamine-induced hepatocellular carcinoma murine model. The results confirmed that NpRg3 administration suppressed the development of lung metastases. Although circulating tumor cells from primary tumors are the main factor affecting cancer metastasis, NpRg3 might inhibit metastatic spreading via the reduced mobilization of endothelial progenitor cells in the circulation [96].

### 4.2. Lung Cancer

Huang et al. observed that bronchial washing fluid might characterize the microbial composition of lung tumor tissues. Higher amounts of *Veillonella*, *Megasphaera*, *Actinomyces*, and *Arthrobacter* genera were documented in a group of lung adenocarcinoma patients without distant metastases than in squamous cell lung carcinoma patients without metastases. However, lower levels of *Capnocytophaga* and *Rothia* were found in lung adenocarcinoma with distant metastases than in squamous cell lung carcinoma with distant metastases. Specifically, the *Streptococcus* genus was more reduced in lung adenocarcinoma with metastases than in lung adenocarcinoma without metastases. The results confirmed that the microbial composition differed in histologic types of lung cancer with or without metastases [97]. Another microbiome study showed higher *Legionella* in lung tissues from patients who developed metastases, while *Thermus* was more abundant in tissue samples from patients with advanced-stage (IIIB and IV) lung cancer [98]. A respiratory tract infected with *Streptococcus pneumoniae* promoted the development of liver metastases in the murine model via TLR2 activation [99]. Higher exposure to cigarette smoke and non-typeable *Haemophilus influenzae* supported lung metastasis growth in the lung cancer model. The authors noted that cigarette smoke caused the disruption of pulmonary barrier integrity, allowing for the translocation of bacterial factors into tumors [100].

### 4.3. Breast Cancer

Some studies highlighted the differences in the microbiome composition between samples from breast tumors and non-malignant breast tissues [101]. For the first time, Buchta Rosean and a research team documented that dysbiotic changes in the gut microbiome increased the dissemination process in the HR+ breast cancer murine model. Antibiotic pretreatment caused changes at the phylum and genus levels. The amounts of *Alistipes*, *Blautia*, *Parabacteroides*, and *Escherichia*/*Shigella* genera were elevated in mice with antibiotic-induced commensal dysbiosis. Antibiotic-induced dysbiosis enhanced tumor cell dissemination in peripheral blood, lungs, and axillary lymph nodes [102]. An analysis of fecal samples revealed that *Acinetobacter*, *Bacilli*, *Campylobacter*, *Collinsella*, *Lactobacillales*, *Veillonella*, *Streptococcus*, *Epsilonproteobacteria*, *Pseudomonadales*, and *Moraxellaceae* were more abundant in the gut microbiome of breast cancer patients with bone metastases than in control samples. However, the analysis revealed significantly decreased amounts of *Megamonas*, *Clostridia*, *Akkermansia*, *Gemmiger*, and *Paraprevotella* in the fecal samples of metastatic patients. Therefore, the authors suggested that lower levels of *Megamonas* and *Akkermansia* might correlate with the presence of bone metastases [103]. The tumor microbiome depletion induced by antibiotic treatment inhibited lung metastasis development in the murine spontaneous breast-tumor model. As detected, the intratumoral administration of specific bacteria, such as *Staphylococcus xylosus*, *Ligilactobacillus animalis*, and *Spilopsyllus cuniculi*, elevated the number of lung metastases without affecting primary tumors in mice [11].

Parhi et al. confirmed the pro-metastatic effect of *F. nucleatum* in the murine model. A tail vein microbe injection supported the progression of AT3 mammary tumors and the development of lung metastases. Mammary tumors were colonized by *Fusobacterium* via bacterial attachment to display Gal-GalNAc on tumor cells [104]. Zhu et al. observed that the administration of broad-spectrum antibiotics promoted cancer metastases and decreased the survival rate, showing low *Bifidobacterium* in antibiotic-treated mice. However, fecal microbiota transplantation (FMT) from SPF mice caused the restoration of the gut microbiome composition [61]. Neoadjuvant chemotherapy decreased the intratumoral level of *Streptococcus* while elevating the amount of *Pseudomonas*. A higher abundance of *Brevundimonas* and *Staphylococcus* was reported in primary breast tumors from women with distant metastases. In vitro experiments showed that breast cancer cells treated with 10% *P. aeruginosa*-conditioned media decreased doxorubicin-mediated cleaved caspase 7 in breast cancer cells [105].

### 4.4. Head and Neck Cancers

Eun et al. documented statistically significant differences in the oral microbiome between patients with and without metastases in oral squamous cell carcinoma. An analysis of the oral microbiome in saliva samples from patients with tumors in the oral cavity showed that *Tannerella* and *Fusobacterium* genera were more abundant in participants without metastases, while *Prevotella*, *Stomatobaculum*, *Bifidobacterium*, *Peptostreptococcaceae*, *Shuttleworthia*, and *Finegoldia* had a higher presence in metastatic patients [106].

A study with 802 nasopharyngeal cancer patients revealed that a higher bacterial load within tumors correlated with worse metastasis-free survival and patient OS. The predominant bacterial taxa in the tumor samples were *Corynebacterium* and *Staphylococcus* [107]. Yuan et al. revealed specific intratumoral bacterial taxa, including *Pseudomonas*, *Rhodococcus*, *Sphingomonas*, *Streptococcus*, *Granulicatella*, *Haemophilus*, and *Coriobacteriales*, which predicted papillary thyroid cancer invasiveness. A higher alpha diversity was documented in tumor samples from patients with advanced stages than in patients with lower disease stages. The interaction between the tumor microbiome and thyroid hormones might contribute to cancer progressiveness, but more research is needed in this area [108].

### 4.5. Genitourinary Cancers

A study of the bacterial composition of fecal samples from patients with advanced metastatic castrate-resistant prostate cancer treated with immunotherapy showed differences between responders and non-responders to pembrolizumab. *Bacteroides thetaiotaomicron*, *B. fragilis*, and *Roseburia unassigned* dominated in responder samples. In contrast to other studies showing that the presence of favorable *Akkermansia muciniphila* correlated with a better anti-cancer treatment response, Peiffer et al. observed its lower amount in responder fecal samples [109]. The level of intratumoral microbes, including *Methylobacterium radiotolerans JCM 2831*, *Delftia acidovorans SPH-1*, *Rhodococcus erythropolis PR4*, *Stenotrophomonas maltophilia K279a*, and *Meiothermus silvanus DSM 9946*, negatively correlated with the tumor-node-metastasis stage in patients with prostate cancer [110].

A higher fecal microbial diversity and *A. muciniphila* enrichment were associated with a better clinical response to nivolumab or nivolumab plus ipilimumab in patients with metastatic renal cell carcinoma [111]. However, an analysis of fecal samples from patients with metastatic renal cell carcinoma noted that *Akkermansia* was not responsible for treatment efficacy due to its detection in the gut microbiome of seven responders and six non-responders. Differences in the baseline gut microbiome between responding and non-responding patients were not supposed to predict patient outcomes [112]. Probiotic supplementation elevated the level of *Bifidobacterium* spp. in patients with metastatic renal cell carcinoma. A clinical benefit from targeted therapy using vascular endothelial growth factor-tyrosine kinase inhibitors was observed in patients enriched with *Barnesiella intestinihominis* and *A. muciniphila* in the microbiome [113]. Antibiotic supplementation targeting *Bacteroides* spp. in the gut microbiome improved progression-free survival (PFS) in patients with metastatic renal cell carcinoma who received VEGF-tyrosine kinase inhibitors (TKIs) [114].

### 4.6. Metastatic Melanoma

Oral and gut microbial communities were diverse in 112 patients with metastatic melanoma. Significant enrichments of *Bacteroidales* in the fecal microbiome and *Lactobacillales* in the oral microbiome were documented in patient samples using 16S rRNA sequencing. In the case of response to programed cell death protein 1 (PD-1) blockade, alpha diversity was higher in the responders’ fecal microbiome, but differences in the oral microbiome were not observed between responders and non-responders to therapy [115]. Mizuhashi et al. found an association between the level of the genus *Corynebacterium* in swab samples and melanoma progression. The results showed that *Corynebacterium* was present in 76.9% and 28.6% of patients at stages III/IV and I/II, respectively [116]. A microbiome analysis of fecal samples from 42 metastatic melanoma patients before immunotherapy uncovered an enrichment of *Bifidobacterium longum*, *Collinsella aerofaciens*, and *E. faecium* in the gut microbiome of responding patients, whereas *Ruminococcus obeum* and *Roseburia intestinalis* were more abundant in non-responders [117]. The gut microbiome of metastatic melanoma patients responding to PD-1 blockade was enriched in *Clostridiales*. On the contrary, a higher amount of *Bacteroidales* dominated in non-responding patients [118]. Vitali et al. found that *Prevotella*, *Clostridium IV*, *Holdemania*, and *Anaerofustis* were abundant in melanoma patients’ gut microbiome. According to the authors, a richer gut microbiome was shown in patients with stage I/II melanoma than in patients with metastatic disease [119].

The fundamental studies focusing on the microbiome composition in metastatic disease are summarized in Table 2.

## 5. Microbiota Modulation and Cancer Progression

Gut microbiome modulation leading to increased intestinal barrier and anti-inflammatory responses might inhibit pro-tumorigenic processes, including cancer progression, migration, invasion, and angiogenesis [120,121]. The intragastric administration of *Lactobacillus reuteri* FLRE5K1 inhibited the incidence of tumors in BALB/C mice injected with melanoma cells. The results indicated that *L. reuteri* FLRE5K1 might restrain the development of tumors due to the blockade of the migration and colonization of cancer cells [122]. A preclinical study noted that fecal transplants from obese mice to lean mice with B16F10 tumors stimulated melanoma development and supported cancer progression [123]. The aim of recent studies is to assess the effect of probiotic supplementation on tumor progression.

Chen et al. showed that probiotics composed of *B. longum*, *Bifidobacterium breve*, *Bifidobacterium infantis*, *Lactobacillus acidophilus*, *Lactobacillus plantarum*, *Lactobacillus casei*, *Lactobacillus bulgaricus*, and *Streptococcus thermophilus* attenuated the development of lung metastases and prolonged survival in melanoma-bearing mice. Probiotic supplementation led to increased levels of *Lachnospiraceae*, *Streptococcus*, and *Lachnoclostridium* [124]. Aerosolized probiotic *Lactobacillus rhamnosus*, which reached lung murine tissue, reduced the number of lung metastases. Moreover, aerosolized *L. rhamnosus* and *Bifidobacterium bifidum* increased the effect of conventional chemotherapeutic dacarbazine in melanoma-bearing mice [125]. A Prohep probiotic product composed of *L. rhamnosus* GG, *E. coli* Nissle 1917, and VSL#3 (1:1:1) orally administered to mice bearing hepatocellular carcinoma retarded tumor growth and inhibited angiogenesis. Prohep administration altered the gut microbiome toward valerate producer *Oscillibacter* and propionate producer *Prevotella*, both known for their ability to reduce Th17 polarization and support Treg/Tr1 cells in the gut. The results showed that probiotics reduced the recruitment of Th17 cells, which secrete pro-inflammatory cytokines to the tumor microenvironment. Additionally, several angiogenic factors, including ANG2, FLT-1, KDR, TEK, and VEGF-A, were downregulated in the probiotic-supplemented group [126]. Daily oral administration of CBM588 containing *Clostridium butyricum* prolonged PFS in patients with metastatic renal cell carcinoma treated with nivolumab and ipilimumab. The level of *Bifidobacterium* spp. was higher in probiotic-supplemented patients who responded to immunotherapy, while the results showed a decline in *Desulfovibrio* spp. However, the toxicity rate was not different between the supplemented and control groups [127]. A probiotic mixture of eight bacterial strains mitigated the length and severity of chemotherapy-associated diarrhea in CRC animal models with liver metastases. Moreover, probiotics support gastrointestinal regeneration after chemotherapeutic treatment [128]. Shang et al. documented that an intragastric probiotic mixture of *B. longum*, *B. bifidum*, *L. acidophilus*, and *L. plantarum* attenuated cancer cell proliferation and even the development of metastasis in mouse models of CRC [129]. Baruch et al. performed FMT from 2 selected donors previously treated with immunotherapy for metastatic melanoma into 10 recipients with confirmed progression on PD–1 blockade. The presence of favorable *Lachnospiraceae* was observed in both donors. The feces transfer from donors caused a shift in the gut microbiome in metastatic melanoma recipients with abundant favorable *Veillonellaceae* and a decline in *B. bifidum* [130].

## 6. Conclusions and Future Directions

The gut and intratumoral microbiomes can influence cancer progression and metastatic processes in various ways, including inducing inflammation and immune system modulation, affecting metabolism and providing energy for cancer cell spread, promoting the angiogenesis caused by microbial metabolites, and impacting cancer treatment efficacy to control metastatic disease. Particular attention should be paid to addressing the ability of specific microorganisms and microbiota-derived metabolites to shape the immune system and tumor microenvironment, potentially promoting the growth and spread of cancer cells. Longitudinal multi-center clinical trials targeting the microbiome composition and function in cancer patients before, during, and after treatment would help uncover distinctive microbiome-associated patterns and their effects on recurrence and survival rates. However, the standardization of methods and procedures for microbiome analyses will be crucial to obtain the most relevant results for clinical use.

Mounting evidence reported a significant correlation between the efficacy of immunotherapy and the gut microbiome composition in patients with metastatic disease. Tumor microbiome profiling along with gut microbiome characterization in individual patients could lead to future personalized therapy to increase the efficacy and decrease the toxicity of anti-cancer treatment. Furthermore, the tumor and gut microbiomes could undergo similar evolution, and the microbiome composition could be assessed at several time points. Another important field of research includes therapeutic interventions aimed at modifying the tumor and/or gut microbiome, including different approaches, such as antibiotic therapy; the modification of the tumor microenvironment; treatments affecting immune cells; and the colonization of beneficial bacteria by prebiotics, probiotics, and fecal microbiome transplant. Further research is required to obtain more evidence elucidating the mechanisms and signaling pathways behind the microbiota–host interactions that drive metastasis and malignant progression. A deeper understanding could help integrate microbiome-based interventions into clinical practice for improving cancer patient outcomes.

## Figures and Tables

**Table 1:** Exploring the microbial markers associated with treatment outcomes in advanced or metastatic cancer patients (according to https://ClinicalTrials.gov/, accessed on 18 October 2023).

Study	Study Design	Disease	Purpose	Patients (n)	Intervention	Study Status
NCT03941080	An observational prospective study	Metastatic CRC	To confirm the microbial taxa associated with treatment response and side effects in metastatic or irresectable disease	300 adults/ older adults	Enrolled patients will be newly diagnosed with an indication for standard palliative systemic treatment.	Recruiting
NCT04579484	An observational prospective study	Metastatic breast cancer	To determine the gut microbiome in fecal samples of patients with ER^+^ HER2^−^ breast cancer and assess the relationship between dietary factors and microbiome	20 adults/ older adults	Patients will receive endocrine therapy with an aromatase inhibitor combined with an inhibitor of cyclin-dependent kinases 4 and 6.	Recruiting
NCT04804956	An observational prospective study	Metastatic rectal cancer	To identify the profile of the mesorectal microbiome and correlation with poor prognosis prediction	100 adults	Participants will receive neoadjuvant treatment.	Recruiting
NCT04579978	An observational prospective study	Metastatic solid cancer	To study changes in the gut microbial community after ICI and evaluate bacterial species associated with treatment efficacy	60 adults/ older adults	Patients will be enrolled in the study for planned standard-of-care ICI.	Recruiting
NCT05878977	An interventional open-label study	Metastatic melanoma	To define novel markers for the prediction of therapy response	150 adults/ older adults	Immunotherapy will consist of PD-1 and CTLA-4 inhibitors.	Recruiting
NCT05635149	An observational prospective study	Metastatic CRC	To assess the composition of the gut microbiome and its association with treatment efficacy	100 adults/ older adults	Patients will be treated with Fruquintinib, ICI plus RT, or Fruquintinib and ICI alone.	Recruiting
NCT05753839	An interventional randomized open-label study with parallel assignment	Metastatic clear cell renal cell carcinoma/kidney cancer	To correlate the gut and urine microbiome compositions with OS, PFS, and ORR	40 adults/ older adults	Patients will receive ICI followed by maintenance therapy with ICI or cytoreductive nephrectomy ± metastasectomy after ICI.	Recruiting
NCT04090710	An interventional randomized study with parallel assignment	Metastatic renal cell carcinoma	To investigate the changes in the gut microbiome via analysis of stool samples	78 children/ adults/ older adults	Patients will undergo cytoreductive stereotactic body RT with a combination of ICIs vs. one ICI alone.	Recruiting
NCT04243720	An observational prospective study	Metastatic solid cancer	To determine changes in the gut microbiome associated with resistance to immunotherapy	100 adults/ older adults	Only participants who progressed on immunotherapy will be enrolled in this study.	Recruiting
NCT04148378	An observational case-only prospective study	CRC neoplasms/ metastatic CRC/ colorectal sarcoma/ adenocarcinoma	To correlate microbiome composition with type of disease	100 children/ adults/ older adults	There is no intervention for the study.	Unknown
NCT04516135	An interventional randomized open-label study with parallel assignment	Metastatic gynecologic cancers	To describe overall diversity, richness, and specific microbial dynamics in the gut and vaginal microbiomes	108 adults/ older adults	Females will be treated with 3D conformal RT/intensity-modulated RT/volume-modulated arc therapy at the physician’s discretion for 1 fraction in the absence of RT-induced toxicities or progression.	Recruiting
NCT04214015	An observational case-only prospective study	Metastatic mesothelioma	To analyze the relative abundance of bacterial members in the gut microbiome	100 children/ adults/ older adults	There is no intervention for the study.	Unknown
NCT03818061	An interventional non-randomized study with parallel assignment	Metastatic HNSCC	To characterize the gut microbiome in immunotherapy using whole-metagenome sequencing	33 adults/ older adults	Patients with/without human papillomavirus will receive atezolizumab combined with bevacizumab.	Active, but not recruiting
NCT03698461	An interventional open-label study with single-group assignment	Metastatic neoplasms/ colorectal neoplasms/ colonic neoplasms/ rectal neoplasms	To determine fecal microbial profile in different time frames	20 adults/ older adults	Anti-cancer treatment will consist of atezolizumab with bevacizumab, levoleucovorin, oxaliplatin, and 5-fluorouracil.	Active, but not recruiting
NCT03977571	An interventional randomized open-label study with parallel assignment	Metastatic renal cell carcinoma/ kidney cancer/ synchronous neoplasm	To correlate the gut microbiome with OS, PFS, and ORR	400 adults/ older adults	Patients will receive deferred cytoreductive nephrectomy/no surgery following nivolumab with ipilimumab or tyrosine kinase inhibitors.	Recruiting
NCT04636775	An observational prospective study	Metastatic non-small-cell lung cancer	To assess the correlations between gut microbiome composition and adverse effects and differences between responders and non-responders	46 adults/ older adults	Patients will be treated with immunotherapy using ICI.	Recruiting
NCT04219137	An observational prospective study	Metastatic EGA	To study the microbiome in feces and rectal swab samples	120 adults/ older adults	Participants will undergo platinum-based chemotherapy.	Unknown
NCT03161756	An interventional non-randomized study parallel assignment	Metastatic melanoma	To explore associations between the gut microbiome and therapy response	72 adults/ older adults	Nivolumab alone or in combination with ipilimumab will be administered intravenously plus denosumab subcutaneously.	Active, but not recruiting
NCT04720768	An interventional open-label study with sequential assignment	Metastatic melanoma	To identify fecal biomarkers associated with therapy response/resistance	78 adults/ older adults	Patients will receive combined treatment with encorafenib, binimetinib, and palbociclib.	Recruiting
NCT03340129	An interventional randomized open-label study with parallel assignment	Metastatic melanoma	To observe the diversity and composition of the gut microbiome and to determine the correlation between mucosal integrity and microbes	218 adults/ older adults	Treatment will include ipilimumab and nivolumab with concurrent intracranial stereotactic RT or ipilimumab and nivolumab alone.	Recruiting

Abbreviations: CRC, colorectal cancer; CTLA-4, cytotoxic T-lymphocyte-associated protein 4; EGA, esophagogastric adenocarcinoma; HNSCC, head and neck squamous cell carcinoma; ICI, immune checkpoint inhibitors; ORR, overall response rate; OS, overall survival; PD-1, programed cell death protein 1; PFS, progression-free survival; RT, radiotherapy.

**Table 2:** Detection of specific microorganisms in advanced/metastatic cancer. The table summarizes fundamental preclinical/clinical studies and their major findings.

Malignancy	Study Type Preclinical/Clinical	Intervention	Changes in Microbial Composition	Major Findings	Ref.
CRC with liver/lung metastases	Patients	Regorafenib plus toripalimab	*Fusobacterium*, *Alistipes*, *Bilophila*, and *Acidaminococcus*	A higher level of specific bacteria was observed in non-responders. Shorter PFS correlated with a higher amount of *Fusobacterium*.	[88]
FAP	Patients/ mice	No intervention provided	*E. coli* and *ETBF*	Both bacterial taxa were biofilm members in FAP tissues from patients. Colonization with *E. coli* and *ETBF* increased DNA damage and IL-17 production in carcinogen-treated mice.	[90]
CRC with liver/lung metastases	Patients	Quxie capsules	Actinobacteria, *Oscillibacter*, *Eubacterium*, and *Lachnospiraceae*	Capsules increased butyrate-producing, immunity-stimulating, and anti-cancer bacterial taxa and enhanced Th cells, both CD4 and CD8 cells.	[93]
PDAC with lymph node metastases	Patients	No intervention	*Leuconostoc*, *Sutterella*, *Comamonas*, and *Turicibacter*	Lower levels of *Leuconostoc* and *Sutterella* were documented in tumors with a size ≥3 cm. An increase in lymph node metastases correlated with a higher abundance of *Comamonas* and *Turicibacter*. On the contrary, *Streptococcus* dominated recurrence-free tumors.	[95]
Hepatocellular carcinoma	Mice	NpRg3	Bacteroidetes, Verrucomicrobia, and Firmicutes	Developed NpRg3 remodeled gut microbiome via reduced Firmicutes and increased Bacteroidetes and Verrucomicrobia in stool samples. Moreover, NpRg3 attenuated tumor development and lung metastatic formation in dimethylnitrosamine-induced spontaneous murine carcinoma.	[96]
Lung cancer	Patients	Systemic therapy/surgical resection	*Legionella* and *Thermus*	*Thermus* was abundant in the lung microbiome in patients with advanced cancer stages, while *Legionella* was enriched in patients with developed metastases. Alpha diversity in tumor tissues was lower than in non-malignant lung tissue samples.	[98]
Hormone receptor-positive breast cancer	Mice	Antibiotic cocktail (vancomycin, ampicillin, metronidazole, neomycin, and gentamicin)	*Blautia*, *Alistipes*, *Blautia*, *Escherichia/Shigella*, and *Bilophila*	Orally gavaged antibiotics caused commensal dysbiosis with a higher abundance of specific genera in poorly metastatic mice. Antibiotics promoted tumor cell dissemination to the lungs/peripheral blood/and lymph nodes.	[102]
Breast cancer	Patients	No intervention	*Streptococcus*, *Campylobacter*, *Moraxellaceae*, *Lactobacillales*, *Bacilli*, *Epsilonproteobacteria*, *Veillonella*, *Acinetobacter*, *Pseudomonadales*, *Megamonas*, and *Akkermansia*	Listed bacteria, except for *Megamonas* and *Akkermansia*, were increased in stool samples of patients with bone metastases. However, the results showed lowered levels of *Megamonas* and *Akkermansia.* Bacterial diversity was reduced in the order of normal controls, patients without metastases, and patients with bone metastases.	[103]
Breast cancer	Patients	Neoadjuvant chemotherapy	*Streptococcus*, *Pseudomonas*, *Brevundimonas*, and *Staphylococcus*	Chemotherapy decreased intratumoral *Streptococcus* and increased *Pseudomonas.* The development of distant metastases correlated with a higher presence of *Brevundimonas* and *Staphylococcus* in primary breast tumors.	[105]
Oral squamous cell carcinoma	Patients	Therapeutic neck dissection due to positive lymph node metastases	*Tannerella*, *Fusobacterium*, *Prevotella*, *Stomatobaculum*, *Bifidobacterium*, *Finegoldia Peptostreptococcaceae*, and *Shuttleworthia*	Two taxa—*Tannerella* and *Fusobacterium*—were enriched in the oral microbiome of patients without metastases. Other genera from the listed panel increased in patients with developed lymph node metastases. Differences in alpha diversity between the oral microbiome of 2 analyzed groups were not significant.	[106]
Castrate-resistant prostate cancer	Patients	Immune checkpoint inhibitor (pembrolizumab)	*A. muciniphila*, *B. thetaiotaomicron*, *B. fragilis*, *and R. unassigned*	*A. muciniphila* was depleted in pembrolizumab responders, while other listed microbes were higher in responding patients.	[109]
Renal cell carcinoma	Patients	Immune checkpoint inhibitor (nivolumab or nivolumab plus ipilimumab	*A. muciniphila*, *B. adolescentis*, *B. intestinihominis*, *Odoribacter splanchnicus*, *Bacteroides ovatus*, and *Eggerthella lenta*	*A. muciniphila*, *B. adolescentis*, *B. intestinihominis*, and *O. splanchnicus* correlated with clinical benefit in metastatic patients, while *B. ovatus* and *E. lenta* were associated with no clinical benefit from immunotherapy.	[111]
Renal cell carcinoma	Patients	Immune checkpoint inhibitor	*Akkermansia*	The presence of *Akkermansia* was documented in both responding and non-responding patients to immunotherapy. Therefore, host-specific or tumor factors might affect therapy response.	[112]
Melanoma	Patients	Immune checkpoint inhibitor	*Lactobacillales*, *Clostridiales/Ruminococcaceae*, *Faecalibacterium*, *Bacteroidales*, *B. thetaiotaomicron*, *E. coli*, and *Anaerotruncus colihominis*	*Lactobacillales* dominated the oral microbiome of all metastatic patients. *Clostridiales/Ruminococcaceae*, *Faecalibacterium*, and alpha diversity were greater in responders, while *Bacteroidales*, *B. thetaiotaomicron*, *E. coli*, and *A. colihominis* were abundant in non-responders.	[115]
Melanoma	Patients	Immune checkpoint inhibitor	*B. longum*, *C. aerofaciens*, *E. faecium*, *R. obeum*, and *R. intestinalis*	The authors observed a higher abundance of *R. obeum* and *R. intestinalis* in non-responders, while the other 3 species were enriched significantly in the responder gut microbiome.	[117]
Melanoma	Patients	Immune checkpoint inhibitor	*Clostridiales* and *Bacteroidales*	Higher bacterial diversity with the prevalence of *Clostridiales* was observed in the gut microbiome of responding patients. However, the dominance of *Bacteroidales* within the gut microbiome characterized non-responders.	[118]
Melanoma	Patients	No intervention	*Corynebacterium*	In swab samples, *Corynebacterium* was the most detected taxa in advanced-stage patients. However, the authors did not detect associations between cutaneous microbiome and cancer stage.	[116]

Abbreviations: ETBF, enterotoxigenic *B. fragilis*; FAP, familial adenomatous polyposis; NpRg3, nanoparticle conjugation of ginsenoside Rg3; PDAC, pancreatic ductal adenocarcinoma; PFS, progression-free survival; Th cells, T helper cells.

## References

[1] Sevcikova A., Izoldova N., Stevurkova V., Kasperova B., Chovanec M., Ciernikova S., Mego M. (2022). The Impact of the Microbiome on Resistance to Cancer Treatment with Chemotherapeutic Agents and Immunotherapy. Int. J. Mol. Sci..

[2] Cullin N., Azevedo Antunes C., Straussman R., Stein-Thoeringer C.K., Elinav E. (2021). Microbiome and cancer. Cancer Cell.

[3] Hanahan D. (2022). Hallmarks of Cancer: New Dimensions. Cancer Discov..

[4] Massague J., Ganesh K. (2021). Metastasis-Initiating Cells and Ecosystems. Cancer Discov..

[5] Gerstberger S., Jiang Q., Ganesh K. (2023). Metastasis. Cell.

[6] Gao F., Yu B., Rao B., Sun Y., Yu J., Wang D., Cui G., Ren Z. (2022). The effect of the intratumoral microbiome on tumor occurrence, progression, prognosis and treatment. Front. Immunol..

[7] Galeano Nino J.L., Wu H., LaCourse K.D., Kempchinsky A.G., Baryiames A., Barber B., Futran N., Houlton J., Sather C., Sicinska E. (2022). Effect of the intratumoral microbiota on spatial and cellular heterogeneity in cancer. Nature.

[8] Liu J., Luo F., Wen L., Zhao Z., Sun H. (2023). Current Understanding of Microbiomes in Cancer Metastasis. Cancers.

[9] Hofman P., Vouret-Craviari V. (2012). Microbes-induced EMT at the crossroad of inflammation and cancer. Gut Microbes.

[10] Caven L.T., Brinkworth A.J., Carabeo R.A. (2023). Chlamydia trachomatis induces the transcriptional activity of host YAP in a Hippo-independent fashion. Front. Cell Infect. Microbiol..

[11] Fu A., Yao B., Dong T., Chen Y., Yao J., Liu Y., Li H., Bai H., Liu X., Zhang Y. (2022). Tumor-resident intracellular microbiota promotes metastatic colonization in breast cancer. Cell.

[12] Brandt S., Kwok T., Hartig R., Konig W., Backert S. (2005). NF-κB activation and potentiation of proinflammatory responses by the *Helicobacter pylori* CagA protein. Proc. Natl. Acad. Sci. USA.

[13] Yin Y., Grabowska A.M., Clarke P.A., Whelband E., Robinson K., Argent R.H., Tobias A., Kumari R., Atherton J.C., Watson S.A. (2010). *Helicobacter pylori* potentiates epithelial:mesenchymal transition in gastric cancer: Links to soluble HB-EGF, gastrin and matrix metalloproteinase-7. Gut.

[14] Fares J., Fares M.Y., Khachfe H.H., Salhab H.A., Fares Y. (2020). Molecular principles of metastasis: A hallmark of cancer revisited. Signal Transduct. Target. Ther..

[15] Thiery J.P. (2002). Epithelial-mesenchymal transitions in tumour progression. Nat. Rev. Cancer.

[16] Yang J., Antin P., Berx G., Blanpain C., Brabletz T., Bronner M., Campbell K., Cano A., Casanova J., Christofori G. (2020). Guidelines and definitions for research on epithelial-mesenchymal transition. Nat. Rev. Mol. Cell Biol..

[17] Giuliano M., Giordano A., Jackson S., Hess K.R., De Giorgi U., Mego M., Handy B.C., Ueno N.T., Alvarez R.H., De Laurentiis M. (2011). Circulating tumor cells as prognostic and predictive markers in metastatic breast cancer patients receiving first-line systemic treatment. Breast Cancer Res..

[18] Mego M., Karaba M., Minarik G., Benca J., Silvia J., Sedlackova T., Manasova D., Kalavska K., Pindak D., Cristofanilli M. (2019). Circulating Tumor Cells with Epithelial-to-mesenchymal Transition Phenotypes Associated with Inferior Outcomes in Primary Breast Cancer. Anticancer Res..

[19] Fridrichova I., Kalinkova L., Ciernikova S. (2022). Clinical Relevancy of Circulating Tumor Cells in Breast Cancer: Epithelial or Mesenchymal Characteristics, Single Cells or Clusters?. Int. J. Mol. Sci..

[20] Kim M.Y., Oskarsson T., Acharyya S., Nguyen D.X., Zhang X.H., Norton L., Massague J. (2009). Tumor self-seeding by circulating cancer cells. Cell.

[21] Comen E., Norton L. (2012). Self-seeding in cancer. Recent Results Cancer Res..

[22] Park J., Wysocki R.W., Amoozgar Z., Maiorino L., Fein M.R., Jorns J., Schott A.F., Kinugasa-Katayama Y., Lee Y., Won N.H. (2016). Cancer cells induce metastasis-supporting neutrophil extracellular DNA traps. Sci. Transl. Med..

[23] Cools-Lartigue J., Spicer J., McDonald B., Gowing S., Chow S., Giannias B., Bourdeau F., Kubes P., Ferri L. (2013). Neutrophil extracellular traps sequester circulating tumor cells and promote metastasis. J. Clin. Investig..

[24] Zhong W., Wang Q., Shen X., Du J. (2023). The emerging role of neutrophil extracellular traps in cancer: From lab to ward. Front. Oncol..

[25] Adrover J.M., McDowell S.A.C., He X.Y., Quail D.F., Egeblad M. (2023). NETworking with cancer: The bidirectional interplay between cancer and neutrophil extracellular traps. Cancer Cell.

[26] Hu W., Lee S.M.L., Bazhin A.V., Guba M., Werner J., Niess H. (2023). Neutrophil extracellular traps facilitate cancer metastasis: Cellular mechanisms and therapeutic strategies. J. Cancer Res. Clin. Oncol..

[27] Nepali P.R., Kyprianou N. (2023). *Anoikis* in phenotypic reprogramming of the prostate tumor microenvironment. Front. Endocrinol..

[28] Paoli P., Giannoni E., Chiarugi P. (2013). *Anoikis* molecular pathways and its role in cancer progression. Biochim. Biophys. Acta (BBA) Mol. Cell Res..

[29] Pretzsch E., Bosch F., Neumann J., Ganschow P., Bazhin A., Guba M., Werner J., Angele M. (2019). Mechanisms of Metastasis in Colorectal Cancer and Metastatic Organotropism: Hematogenous versus Peritoneal Spread. J. Oncol..

[30] Peinado H., Zhang H., Matei I.R., Costa-Silva B., Hoshino A., Rodrigues G., Psaila B., Kaplan R.N., Bromberg J.F., Kang Y. (2017). Pre-metastatic niches: Organ-specific homes for metastases. Nat. Rev. Cancer.

[31] Kaplan R.N., Riba R.D., Zacharoulis S., Bramley A.H., Vincent L., Costa C., MacDonald D.D., Jin D.K., Shido K., Kerns S.A. (2005). VEGFR1-positive haematopoietic bone marrow progenitors initiate the pre-metastatic niche. Nature.

[32] Huang H. (2018). Matrix Metalloproteinase-9 (MMP-9) as a Cancer Biomarker and MMP-9 Biosensors: Recent Advances. Sensors.

[33] Kryczek I., Wei S., Keller E., Liu R., Zou W. (2007). Stroma-derived factor (SDF-1/CXCL12) and human tumor pathogenesis. Am. J. Physiol. Cell Physiol..

[34] Xu C., Zhao H., Chen H., Yao Q. (2015). CXCR4 in breast cancer: Oncogenic role and therapeutic targeting. Drug Des. Dev. Ther..

[35] Muller A., Homey B., Soto H., Ge N., Catron D., Buchanan M.E., McClanahan T., Murphy E., Yuan W., Wagner S.N. (2001). Involvement of chemokine receptors in breast cancer metastasis. Nature.

[36] Kaplan R.N., Rafii S., Lyden D. (2006). Preparing the “soil”: The premetastatic niche. Cancer Res..

[37] De Palma M., Biziato D., Petrova T.V. (2017). Microenvironmental regulation of tumour angiogenesis. Nat. Rev. Cancer.

[38] Kim H.J., Ji Y.R., Lee Y.M. (2022). Crosstalk between angiogenesis and immune regulation in the tumor microenvironment. Arch. Pharm. Res..

[39] Zirlik K., Duyster J. (2018). Anti-Angiogenics: Current Situation and Future Perspectives. Oncol. Res. Treat..

[40] Ornitz D.M., Itoh N. (2015). The Fibroblast Growth Factor signaling pathway. Wiley Interdiscip. Rev. Dev. Biol..

[41] Harry J.A., Ormiston M.L. (2021). Novel Pathways for Targeting Tumor Angiogenesis in Metastatic Breast Cancer. Front. Oncol..

[42] Olejarz W., Kubiak-Tomaszewska G., Chrzanowska A., Lorenc T. (2020). Exosomes in Angiogenesis and Anti-Angiogenic Therapy in Cancers. Int. J. Mol. Sci..

[43] Roche J. (2018). The Epithelial-to-Mesenchymal Transition in Cancer. Cancers.

[44] Umar S. (2014). Enteric pathogens and cellular transformation: Bridging the gaps. Oncotarget.

[45] Thiery J.P., Acloque H., Huang R.Y., Nieto M.A. (2009). Epithelial-mesenchymal transitions in development and disease. Cell.

[46] Chandrakesan P., Roy B., Jakkula L.U., Ahmed I., Ramamoorthy P., Tawfik O., Papineni R., Houchen C., Anant S., Umar S. (2014). Utility of a bacterial infection model to study epithelial-mesenchymal transition, mesenchymal-epithelial transition or tumorigenesis. Oncogene.

[47] Basu M., Philipp L.M., Baines J.F., Sebens S. (2021). The Microbiome Tumor Axis: How the Microbiome Could Contribute to Clonal Heterogeneity and Disease Outcome in Pancreatic Cancer. Front. Oncol..

[48] Wan G., Xie M., Yu H., Chen H. (2018). Intestinal dysbacteriosis activates tumor-associated macrophages to promote epithelial-mesenchymal transition of colorectal cancer. Innate Immun..

[49] Henstra C., van Praagh J., Olinga P., Nagelkerke A. (2021). The gastrointestinal microbiota in colorectal cancer cell migration and invasion. Clin. Exp. Metastasis.

[50] Wang Q., Yu C., Yue C., Liu X. (2020). *Fusobacterium nucleatum* produces cancer stem cell characteristics via EMT-resembling variations. Int. J. Clin. Exp. Pathol..

[51] Baud J., Varon C., Chabas S., Chambonnier L., Darfeuille F., Staedel C. (2013). *Helicobacter pylori* initiates a mesenchymal transition through ZEB1 in gastric epithelial cells. PLoS ONE.

[52] Marques M.S., Melo J., Cavadas B., Mendes N., Pereira L., Carneiro F., Figueiredo C., Leite M. (2018). Afadin Downregulation by *Helicobacter pylori* Induces Epithelial to Mesenchymal Transition in Gastric Cells. Front. Microbiol..

[53] Li W.T., Iyangar A.S., Reddy R., Chakladar J., Bhargava V., Sakamoto K., Ongkeko W.M., Rajasekaran M. (2021). The Bladder Microbiome Is Associated with Epithelial-Mesenchymal Transition in Muscle Invasive Urothelial Bladder Carcinoma. Cancers.

[54] Abd-El-Raouf R., Ouf S.A., Gabr M.M., Zakaria M.M., El-Yasergy K.F., Ali-El-Dein B. (2020). Escherichia coli foster bladder cancer cell line progression via epithelial mesenchymal transition, stemness and metabolic reprogramming. Sci. Rep..

[55] Katz J., Onate M.D., Pauley K.M., Bhattacharyya I., Cha S. (2011). Presence of *Porphyromonas gingivalis* in gingival squamous cell carcinoma. Int. J. Oral. Sci..

[56] Abdulkareem A.A., Shelton R.M., Landini G., Cooper P.R., Milward M.R. (2018). Periodontal pathogens promote epithelial-mesenchymal transition in oral squamous carcinoma cells in vitro. Cell Adhes. Migr..

[57] Ha N.H., Woo B.H., Kim D.J., Ha E.S., Choi J.I., Kim S.J., Park B.S., Lee J.H., Park H.R. (2015). Prolonged and repetitive exposure to *Porphyromonas gingivalis* increases aggressiveness of oral cancer cells by promoting acquisition of cancer stem cell properties. Tumour Biol..

[58] Sztukowska M.N., Ojo A., Ahmed S., Carenbauer A.L., Wang Q., Shumway B., Jenkinson H.F., Wang H., Darling D.S., Lamont R.J. (2016). *Porphyromonas gingivalis* initiates a mesenchymal-like transition through ZEB1 in gingival epithelial cells. Cell Microbiol..

[59] Peng Z., Wan P., Deng Y., Shen W., Liu R. (2023). Lipopolysaccharide exacerbates to the migration, invasion, and epithelial-mesenchymal transition of esophageal cancer cells by TLR4/NF-κB axis. Environ. Toxicol..

[60] Nikolaieva N., Sevcikova A., Omelka R., Martiniakova M., Mego M., Ciernikova S. (2022). Gut Microbiota-MicroRNA Interactions in Intestinal Homeostasis and Cancer Development. Microorganisms.

[61] Zhu Z., Huang J., Li X., Xing J., Chen Q., Liu R., Hua F., Qiu Z., Song Y., Bai C. (2020). Gut microbiota regulate tumor metastasis via circRNA/miRNA networks. Gut Microbes.

[62] Yan X., Liu L., Li H., Qin H., Sun Z. (2017). Clinical significance of *Fusobacterium nucleatum*, epithelial-mesenchymal transition, and cancer stem cell markers in stage III/IV colorectal cancer patients. OncoTargets Ther..

[63] Yang Y., Weng W., Peng J., Hong L., Yang L., Toiyama Y., Gao R., Liu M., Yin M., Pan C. (2017). *Fusobacterium nucleatum* Increases Proliferation of Colorectal Cancer Cells and Tumor Development in Mice by Activating Toll-Like Receptor 4 Signaling to Nuclear Factor-κB, and Up-regulating Expression of MicroRNA-21. Gastroenterology.

[64] Kovacs T., Miko E., Ujlaki G., Yousef H., Csontos V., Uray K., Bai P. (2021). The involvement of oncobiosis and bacterial metabolite signaling in metastasis formation in breast cancer. Cancer Metastasis Rev..

[65] Ujlaki G., Kovacs T., Vida A., Kokai E., Rauch B., Schwarcz S., Miko E., Janka E., Sipos A., Hegedus C. (2023). Identification of Bacterial Metabolites Modulating Breast Cancer Cell Proliferation and Epithelial-Mesenchymal Transition. Molecules.

[66] Sari Z., Miko E., Kovacs T., Janko L., Csonka T., Lente G., Sebo E., Toth J., Toth D., Arkosy P. (2020). Indolepropionic Acid, a Metabolite of the Microbiome, Has Cytostatic Properties in Breast Cancer by Activating AHR and PXR Receptors and Inducing Oxidative Stress. Cancers.

[67] Wang D., Cheng J., Zhang J., Zhou F., He X., Shi Y., Tao Y. (2021). The Role of Respiratory Microbiota in Lung Cancer. Int. J. Biol. Sci..

[68] Castro P.R., Bittencourt L.F.F., Larochelle S., Andrade S.P., Mackay C.R., Slevin M., Moulin V.J., Barcelos L.S. (2021). GPR43 regulates sodium butyrate-induced angiogenesis and matrix remodeling. Am. J. Physiol. Heart Circ. Physiol..

[69] Nguyen T.T., Lian S., Ung T.T., Xia Y., Han J.Y., Jung Y.D. (2017). Lithocholic Acid Stimulates IL-8 Expression in Human Colorectal Cancer Cells via Activation of Erk1/2 MAPK and Suppression of STAT3 Activity. J. Cell. Biochem..

[70] Saha S., Chowdhury P., Pal A., Chakrabarti M.K. (2008). Downregulation of human colon carcinoma cell (COLO-205) proliferation through PKG-MAP kinase mediated signaling cascade by *E. coli* heat stable enterotoxin (STa), a potent anti-angiogenic and anti-metastatic molecule. J. Appl. Toxicol..

[71] Yang S., Dai H., Lu Y., Li R., Gao C., Pan S. (2022). Trimethylamine N-Oxide Promotes Cell Proliferation and Angiogenesis in Colorectal Cancer. J. Immunol. Res..

[72] Salahshouri P., Emadi-Baygi M., Jalili M., Khan F.M., Wolkenhauer O., Salehzadeh-Yazdi A. (2021). A Metabolic Model of Intestinal Secretions: The Link between Human Microbiota and Colorectal Cancer Progression. Metabolites.

[73] Wu J., Li H., Xie H., Wu X., Lan P. (2019). The malignant role of exosomes in the communication among colorectal cancer cell, macrophage and microbiome. Carcinogenesis.

[74] Wong S.H., Zhao L., Zhang X., Nakatsu G., Han J., Xu W., Xiao X., Kwong T.N.Y., Tsoi H., Wu W.K.K. (2017). Gavage of Fecal Samples from Patients with Colorectal Cancer Promotes Intestinal Carcinogenesis in Germ-Free and Conventional Mice. Gastroenterology.

[75] De Spiegeleer B., Verbeke F., D’Hondt M., Hendrix A., Van De Wiele C., Burvenich C., Peremans K., De Wever O., Bracke M., Wynendaele E. (2015). The quorum sensing peptides PhrG, CSP and EDF promote angiogenesis and invasion of breast cancer cells in vitro. PLoS ONE.

[76] Wynendaele E., Verbeke F., D’Hondt M., Hendrix A., Van De Wiele C., Burvenich C., Peremans K., De Wever O., Bracke M., De Spiegeleer B. (2015). Crosstalk between the microbiome and cancer cells by quorum sensing peptides. Peptides.

[77] Hilmi M., Kamal M., Vacher S., Dupain C., Ibadioune S., Halladjian M., Sablin M.P., Marret G., Ajgal Z.C., Nijnikoff M. (2023). Intratumoral microbiome is driven by metastatic site and associated with immune histopathological parameters: An ancillary study of the SHIVA clinical trial. Eur. J. Cancer.

[78] Hajjar J., Mendoza T., Zhang L., Fu S., Piha-Paul S.A., Hong D.S., Janku F., Karp D.D., Ballhausen A., Gong J. (2021). Associations between the gut microbiome and fatigue in cancer patients. Sci. Rep..

[79] Sethi V., Kurtom S., Tarique M., Lavania S., Malchiodi Z., Hellmund L., Zhang L., Sharma U., Giri B., Garg B. (2018). Gut Microbiota Promotes Tumor Growth in Mice by Modulating Immune Response. Gastroenterology.

[80] Spakowicz D., Hoyd R., Muniak M., Husain M., Bassett J.S., Wang L., Tinoco G., Patel S.H., Burkart J., Miah A. (2020). Inferring the role of the microbiome on survival in patients treated with immune checkpoint inhibitors: Causal modeling, timing, and classes of concomitant medications. BMC Cancer.

[81] Dzutsev A., Goldszmid R.S., Viaud S., Zitvogel L., Trinchieri G. (2015). The role of the microbiota in inflammation, carcinogenesis, and cancer therapy. Eur. J. Immunol..

[82] Wang T., Cai G., Qiu Y., Fei N., Zhang M., Pang X., Jia W., Cai S., Zhao L. (2012). Structural segregation of gut microbiota between colorectal cancer patients and healthy volunteers. ISME J..

[83] Viljoen K.S., Dakshinamurthy A., Goldberg P., Blackburn J.M. (2015). Quantitative profiling of colorectal cancer-associated bacteria reveals associations between *Fusobacterium* spp., enterotoxigenic Bacteroides fragilis (ETBF) and clinicopathological features of colorectal cancer. PLoS ONE.

[84] Xu C., Fan L., Lin Y., Shen W., Qi Y., Zhang Y., Chen Z., Wang L., Long Y., Hou T. (2021). *Fusobacterium nucleatum* promotes colorectal cancer metastasis through miR-1322/CCL20 axis and M2 polarization. Gut Microbes.

[85] Chen S., Su T., Zhang Y., Lee A., He J., Ge Q., Wang L., Si J., Zhuo W., Wang L. (2020). *Fusobacterium nucleatum* promotes colorectal cancer metastasis by modulating KRT7-AS/KRT7. Gut Microbes.

[86] Chen S., Zhang L., Li M., Zhang Y., Sun M., Wang L., Lin J., Cui Y., Chen Q., Jin C. (2022). *Fusobacterium nucleatum* reduces METTL3-mediated m^6^A modification and contributes to colorectal cancer metastasis. Nat. Commun..

[87] Bullman S., Pedamallu C.S., Sicinska E., Clancy T.E., Zhang X., Cai D., Neuberg D., Huang K., Guevara F., Nelson T. (2017). Analysis of *Fusobacterium* persistence and antibiotic response in colorectal cancer. Science.

[88] Wang F., He M.M., Yao Y.C., Zhao X., Wang Z.Q., Jin Y., Luo H.Y., Li J.B., Wang F.H., Qiu M.Z. (2021). Regorafenib plus toripalimab in patients with metastatic colorectal cancer: A phase Ib/II clinical trial and gut microbiome analysis. Cell Rep. Med..

[89] Ambalam P., Raman M., Purama R.K., Doble M. (2016). Probiotics, prebiotics and colorectal cancer prevention. Best Pract. Res. Clin. Gastroenterol..

[90] Dejea C.M., Fathi P., Craig J.M., Boleij A., Taddese R., Geis A.L., Wu X., DeStefano Shields C.E., Hechenbleikner E.M., Huso D.L. (2018). Patients with familial adenomatous polyposis harbor colonic biofilms containing tumorigenic bacteria. Science.

[91] Stakelum A., Zaborowski A., Collins D., Winter D.C. (2020). The influence of the gastrointestinal microbiome on colorectal metastasis: A narrative review. Colorectal Dis..

[92] Wachsmannova L., Stevurkova V., Ciernikova S. (2019). Changes in SNAI1 and VIM gene expression in Caco2 cells after cocultivation with bacteria from colorectal cancer biopsies. Neoplasma.

[93] Sun L., Yan Y., Chen D., Yang Y. (2020). Quxie Capsule Modulating Gut Microbiome and Its Association with T cell Regulation in Patients with Metastatic Colorectal Cancer: Result From a Randomized Controlled Clinical Trial. Integr. Cancer Ther..

[94] Li J.J., Zhu M., Kashyap P.C., Chia N., Tran N.H., McWilliams R.R., Bekaii-Saab T.S., Ma W.W. (2021). The role of microbiome in pancreatic cancer. Cancer Metastasis Rev..

[95] Jeong J.Y., Kim T.B., Kim J., Choi H.W., Kim E.J., Yoo H.J., Lee S., Jun H.R., Yoo W., Kim S. (2020). Diversity in the Extracellular Vesicle-Derived Microbiome of Tissues according to Tumor Progression in Pancreatic Cancer. Cancers.

[96] Ren Z., Chen X., Hong L., Zhao X., Cui G., Li A., Liu Y., Zhou L., Sun R., Shen S. (2020). Nanoparticle Conjugation of Ginsenoside Rg3 Inhibits Hepatocellular Carcinoma Development and Metastasis. Small.

[97] Huang D., Su X., Yuan M., Zhang S., He J., Deng Q., Qiu W., Dong H., Cai S. (2019). The characterization of lung microbiome in lung cancer patients with different clinicopathology. Am. J. Cancer Res..

[98] Yu G., Gail M.H., Consonni D., Carugno M., Humphrys M., Pesatori A.C., Caporaso N.E., Goedert J.J., Ravel J., Landi M.T. (2016). Characterizing human lung tissue microbiota and its relationship to epidemiological and clinical features. Genome Biol..

[99] Gowing S.D., Chow S.C., Cools-Lartigue J.J., Chen C.B., Najmeh S., Jiang H.Y., Bourdeau F., Beauchamp A., Mancini U., Angers I. (2017). Gram-positive pneumonia augments non-small cell lung cancer metastasis via host toll-like receptor 2 activation. Int. J. Cancer.

[100] Jungnickel C., Wonnenberg B., Karabiber O., Wolf A., Voss M., Wolf L., Honecker A., Kamyschnikow A., Herr C., Bals R. (2015). Cigarette smoke-induced disruption of pulmonary barrier and bacterial translocation drive tumor-associated inflammation and growth. Am. J. Physiol. Lung Cell Mol. Physiol..

[101] Hadzega D., Minarik G., Karaba M., Kalavska K., Benca J., Ciernikova S., Sedlackova T., Nemcova P., Bohac M., Pindak D. (2021). Uncovering Microbial Composition in Human Breast Cancer Primary Tumour Tissue Using Transcriptomic RNA-seq. Int. J. Mol. Sci..

[102] Buchta Rosean C., Bostic R.R., Ferey J.C.M., Feng T.Y., Azar F.N., Tung K.S., Dozmorov M.G., Smirnova E., Bos P.D., Rutkowski M.R. (2019). Preexisting Commensal Dysbiosis Is a Host-Intrinsic Regulator of Tissue Inflammation and Tumor Cell Dissemination in Hormone Receptor-Positive Breast Cancer. Cancer Res..

[103] Wenhui Y., Zhongyu X., Kai C., Zhaopeng C., Jinteng L., Mengjun M., Zepeng S., Yunshu C., Peng W., Yanfeng W. (2022). Variations in the Gut Microbiota in Breast Cancer Occurrence and Bone Metastasis. Front. Microbiol..

[104] Parhi L., Alon-Maimon T., Sol A., Nejman D., Shhadeh A., Fainsod-Levi T., Yajuk O., Isaacson B., Abed J., Maalouf N. (2020). Breast cancer colonization by *Fusobacterium nucleatum* accelerates tumor growth and metastatic progression. Nat. Commun..

[105] Chiba A., Bawaneh A., Velazquez C., Clear K.Y.J., Wilson A.S., Howard-McNatt M., Levine E.A., Levi-Polyachenko N., Yates-Alston S.A., Diggle S.P. (2020). Neoadjuvant Chemotherapy Shifts Breast Tumor Microbiota Populations to Regulate Drug Responsiveness and the Development of Metastasis. Mol. Cancer Res..

[106] Eun Y.G., Lee J.W., Kim S.W., Hyun D.W., Bae J.W., Lee Y.C. (2021). Oral microbiome associated with lymph node metastasis in oral squamous cell carcinoma. Sci. Rep..

[107] Qiao H., Tan X.R., Li H., Li J.Y., Chen X.Z., Li Y.Q., Li W.F., Tang L.L., Zhou G.Q., Zhang Y. (2022). Association of Intratumoral Microbiota with Prognosis in Patients with Nasopharyngeal Carcinoma from 2 Hospitals in China. JAMA Oncol..

[108] Yuan L., Yang P., Wei G., Hu X., Chen S., Lu J., Yang L., He X., Bao G. (2022). Tumor microbiome diversity influences papillary thyroid cancer invasion. Commun. Biol..

[109] Peiffer L.B., White J.R., Jones C.B., Slottke R.E., Ernst S.E., Moran A.E., Graff J.N., Sfanos K.S. (2022). Composition of gastrointestinal microbiota in association with treatment response in individuals with metastatic castrate resistant prostate cancer progressing on enzalutamide and initiating treatment with anti-PD-1 (pembrolizumab). Neoplasia.

[110] Ma J., Gnanasekar A., Lee A., Li W.T., Haas M., Wang-Rodriguez J., Chang E.Y., Rajasekaran M., Ongkeko W.M. (2020). Influence of Intratumor Microbiome on Clinical Outcome and Immune Processes in Prostate Cancer. Cancers.

[111] Salgia N.J., Bergerot P.G., Maia M.C., Dizman N., Hsu J., Gillece J.D., Folkerts M., Reining L., Trent J., Highlander S.K. (2020). Stool Microbiome Profiling of Patients with Metastatic Renal Cell Carcinoma Receiving Anti-PD-1 Immune Checkpoint Inhibitors. Eur. Urol..

[112] Agarwal A., Modliszewski J., Davey L., Reyes-Martinez M., Runyambo D., Corcoran D., Dressman H., George D.J., Valdivia R.H., Armstrong A.J. (2020). Investigating the role of the gastrointestinal microbiome in response to immune checkpoint inhibitors (ICIs) among patients (pts) with metastatic renal cell carcinoma (mRCC). J. Clin. Oncol..

[113] Dizman N., Hsu J., Bergerot P.G., Gillece J.D., Folkerts M., Reining L., Trent J., Highlander S.K., Pal S.K. (2021). Randomized trial assessing impact of probiotic supplementation on gut microbiome and clinical outcome from targeted therapy in metastatic renal cell carcinoma. Cancer Med..

[114] Hahn A.W., Froerer C., VanAlstine S., Rathi N., Bailey E.B., Stenehjem D.D., Agarwal N. (2018). Targeting Bacteroides in Stool Microbiome and Response to Treatment with First-Line VEGF Tyrosine Kinase Inhibitors in Metastatic Renal-Cell Carcinoma. Clin. Genitourin. Cancer.

[115] Gopalakrishnan V., Spencer C.N., Nezi L., Reuben A., Andrews M.C., Karpinets T.V., Prieto P.A., Vicente D., Hoffman K., Wei S.C. (2018). Gut microbiome modulates response to anti-PD-1 immunotherapy in melanoma patients. Science.

[116] Mizuhashi S., Kajihara I., Sawamura S., Kanemaru H., Makino K., Aoi J., Makino T., Masuguchi S., Fukushima S., Ihn H. (2021). Skin microbiome in acral melanoma: *Corynebacterium* is associated with advanced melanoma. J. Dermatol..

[117] Matson V., Fessler J., Bao R., Chongsuwat T., Zha Y., Alegre M.L., Luke J.J., Gajewski T.F. (2018). The commensal microbiome is associated with anti-PD-1 efficacy in metastatic melanoma patients. Science.

[118] Wargo J.A., Gopalakrishnan V., Spencer C., Karpinets T., Reuben A., Andrews M.C., Tetzlaff M.T., Lazar A., Hwu P., Hwu W.J. (2017). Association of the diversity and composition of the gut microbiome with responses and survival (PFS) in metastatic melanoma (MM) patients (pts) on anti-PD-1 therapy. J. Clin. Oncol..

[119] Vitali F., Colucci R., Di Paola M., Pindo M., De Filippo C., Moretti S., Cavalieri D. (2022). Early melanoma invasivity correlates with gut fungal and bacterial profiles. Br. J. Dermatol..

[120] Ciernikova S., Mego M., Hainova K., Adamcikova Z., Stevurkova V., Zajac V. (2015). Modification of microflora imbalance: Future directions for prevention and treatment of colorectal cancer?. Neoplasma.

[121] Wang Z., Li L., Wang S., Wei J., Qu L., Pan L., Xu K. (2022). The role of the gut microbiota and probiotics associated with microbial metabolisms in cancer prevention and therapy. Front. Pharmacol..

[122] Luo M., Hu M., Xu F., Wu X., Dong D., Wang W. (2020). Preventive effect of Lactobacillus reuteri on melanoma. Biomed. Pharmacother..

[123] Pereira F.V., Melo A.C.L., Silva M.B., de Melo F.M., Terra F.F., Castro I.A., Perandini L.A., Miyagi M.T., Sato F.T., Origassa C.S.T. (2021). Interleukin-6 and the Gut Microbiota Influence Melanoma Progression in Obese Mice. Nutr. Cancer.

[124] Chen L., Zhou X., Wang Y., Wang D., Ke Y., Zeng X. (2021). Propionate and Butyrate Produced by Gut Microbiota after Probiotic Supplementation Attenuate Lung Metastasis of Melanoma Cells in Mice. Mol. Nutr. Food Res..

[125] Le Noci V., Guglielmetti S., Arioli S., Camisaschi C., Bianchi F., Sommariva M., Storti C., Triulzi T., Castelli C., Balsari A. (2018). Modulation of Pulmonary Microbiota by Antibiotic or Probiotic Aerosol Therapy: A Strategy to Promote Immunosurveillance against Lung Metastases. Cell Rep..

[126] Li J., Sung C.Y., Lee N., Ni Y., Pihlajamaki J., Panagiotou G., El-Nezami H. (2016). Probiotics modulated gut microbiota suppresses hepatocellular carcinoma growth in mice. Proc. Natl. Acad. Sci. USA.

[127] Dizman N., Meza L., Bergerot P., Alcantara M., Dorff T., Lyou Y., Frankel P., Cui Y., Mira V., Llamas M. (2022). Nivolumab plus ipilimumab with or without live bacterial supplementation in metastatic renal cell carcinoma: A randomized phase 1 trial. Nat. Med..

[128] Jakubauskas M., Jakubauskiene L., Leber B., Horvath A., Strupas K., Stiegler P., Schemmer P. (2023). Probiotic Supplementation Attenuates Chemotherapy-Induced Intestinal Mucositis in an Experimental Colorectal Cancer Liver Metastasis Rat Model. Nutrients.

[129] Shang F., Jiang X., Wang H., Chen S., Wang X., Liu Y., Guo S., Li D., Yu W., Zhao Z. (2020). The inhibitory effects of probiotics on colon cancer cells: In vitro and in vivo studies. J. Gastrointest. Oncol..

[130] Baruch E.N., Youngster I., Ben-Betzalel G., Ortenberg R., Lahat A., Katz L., Adler K., Dick-Necula D., Raskin S., Bloch N. (2021). Fecal microbiota transplant promotes response in immunotherapy-refractory melanoma patients. Science.

